# Mature Ovarian Teratoma with Carcinoid Tumor in a 28-Year-Old Patient

**DOI:** 10.1155/2013/108582

**Published:** 2013-07-25

**Authors:** Stamatios Petousis, Ioannis Kalogiannidis, Chrysoula Margioula-Siarkou, Alexandros Traianos, Dimosthenis Miliaras, Apostolos Kamparoudis, Apostolos Mamopoulos, David Rousso

**Affiliations:** ^1^3rd Department of Obstetrics and Gynaecology, Aristotle University of Thessaloniki, Konstantinoupoleos 49, 54642, Greece; ^2^Laboratory of Histology, Aristotle University of Thessaloniki, 54124 Panemistimioupolis, Greece; ^3^5th Surgical Department, Aristotle University of Thessaloniki, 54124 Panemistimioupolis, Greece

## Abstract

*Introduction*. Coexistence of carcinoid tumor inside a mature cystic teratoma is an extremely rare phenomenon, especially in young women. We present the case of a 28-year-old woman diagnosed with a right ovarian carcinoid and treated uneventfully with conservative surgical approach. *Case Report*. A 28-year-old woman, gravid 0, parity 0, presented to our department for her annual gynecological examination and Pap smear test. During her examination, a mobile cystic mass was detected in the right lower abdomen. Ultrasound indicated a right ovarian mass 10.5 × 6.3 cm, confirmed by CT scan. Further investigation revealed AFP levels (1539 ng/mL). The ovarian mass was excised by laparoscopy, leaving intact the remaining right ovary. Frozen sections showed a mature cystic teratoma. However, paraffin sections revealed the presence of a small carcinoid within the teratoma's gastric-type mucosa. The patient was set to a close followup. Nine months postoperatively, ultrasound pelvis imaging and CT scan of the abdomen as well as serum tumor markers have shown no evidence of recurrence disease. *Conclusion*. Despite the weak evidence, fertility spare surgical approach for women wanting to preserve their genital tract might be a reasonable option.

## 1. Introduction

Mature cystic teratomas (MCT) represent 10–20% of all ovarian neoplasms [1]. They mainly present in young women, and by definition they are characterized by benign histologic features. Malignant transformation of teratoma (TMT), predominantly to squamous cell carcinomas, may be observed in 1–3% of mature teratomas [2]. However, despite the relatively low incidence of malignant transformation, a number of other malignant tumors arising from MCT have been reported, including adenocarcinoma, thyroid carcinoma, sebaceous carcinoma, malignant melanoma, and sarcoma, while cases with metastatic behavior have also been reported [3–6]. 

The occasion of a carcinoid tumor arising from MCT is very rare with a very small number of published cases [7, 8]. Furthermore, patients with TMT are at least 15 years older than the average patient with mature cystic teratoma and the majority of reports concern postmenopausal women [9]. Therefore, the coexistence of a carcinoid tumor inside a mature ovarian cystic teratoma in young patients is an extremely rare phenomenon of high clinical interest that poses a challenging dilemma about the optimal therapeutic strategy, especially for women desiring to preserve their fertility. 

We present a case of a 28-year-old nulliparous woman diagnosed with carcinoid tumor arising from a mature ovarian cystic teratoma. 

## 2. Case Report 

A 28-year-old woman, gravid 0, parity 0, presented to our department for her annual gynecological examination and Pap smear test. The woman had an uncomplicated medical and gynecological history with regular menstrual cycle. 

During her examination, a mobile cystic mass was detected in the right lower abdomen. Ultrasound examination confirmed the presence of a mass approximately 10 × 6 cm derived from the right ovary, characterized by echogenic heterogeneity. Further investigation of the patient was decided, including serum tumor markers (CEA, CA 9-19, CA 125, AFP, b-HCG) and computed tomography (CT) of the abdomen. All serum markers were normal with the exception of AFP (1539 ng/mL). CT scan confirmed the presence of a right ovarian mass (10.5 × 6.3 cm) containing calcification, while no other peritoneal pathology was demonstrated. 

The right ovarian mass was excised by laparoscopy, leaving intact the remaining right ovary. Frozen sections showed a mature cystic teratoma and so no other intervention was performed at the time of the operation. 

Increased AFP levels returned postoperatively to normal and the woman was discharged the next day without any remarkable complication being mentioned. 

**Figure 1:**
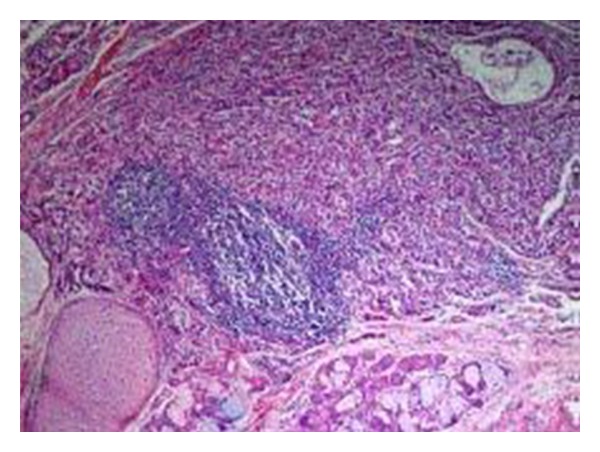
A carcinoid tumor is seen in the upper half of the picture. An island of mature hyaline cartilage (lower left), and lobules of mucous and serous glands (lower part towards the center) are also seen (H&E, ×100).

**Figure 2:**
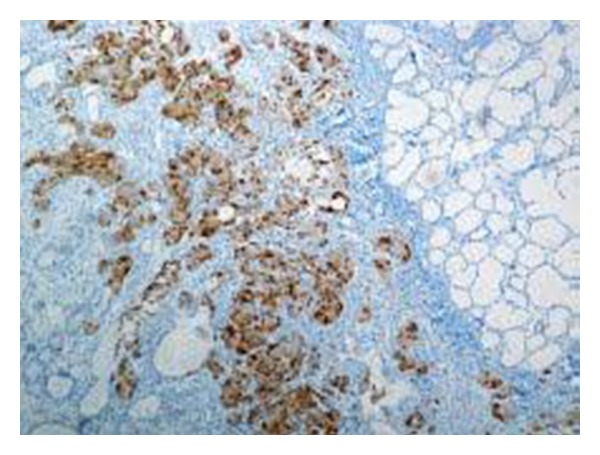
Carcinoid tumor cells showing positive reaction to chromogranin (DAB/Haematoxylin, ×100).

However, paraffin sections revealed a mature teratoma that presented an incidental microscopic focus of a carcinoid tumor, 5 mm in diameter, not seen in the frozen sections. The carcinoid consisted of trabeculae and groups of cells with mild nuclear atypia (Figure 1), which were chromogranin and CD56 positive by immunohistochemistry (Figure 2). The lesion was localized in an area of gastric-type mucosa of the teratoma, which also included epidermal-type squamous epithelium lining the cyst, sebaceous glands, sweat glands, mucous and serous glands, hyaline cartilage, and mature nervous tissue. 

The weak evidence regarding the optimal management of a carcinoid arising from an MCT, the small size of the lesion, which was confined to the gastric mucosa within the mass of the MCT, and the fact that our patient was young and nulliparous led us not to proceed in further radical operation. The patient was set to a close followup with gynaecological examination and ultrasound imaging every 3 months and performance of CT scan every 6 months. Conservative approach and close followup were also considered as the optimal approach by the attending surgeon. Nine months postoperatively, ultrasound pelvis imaging and CT scan of the abdomen as well as serum tumor markers have shown no evidence of recurrent disease. 

## 3. Discussion 

Teratomas represent neoplasms containing cells of multiple germ cell layers that may be observed in several anatomic regions, including ovary, testis, peritoneum, spinal cord, or kidney [10, 11]. The coexistence of a malignancy within a mature teratoma, as in the present case, is often reported as “teratoma with malignant transformation” (TMT) [10]. TMT is an extremely rare phenomenon, being reported only in 1–3% of the teratomas, while in the majority of these cases, the predominant histological type of coexisting tumor is squamous cell carcinoma. Growth of carcinoid within a teratoma is even rarer, representing almost 5% of cases with TMT [12]. In addition, patients with TMT are older (almost 15 years) than the patients with mature cystic teratoma and the majority of reports concern postmenopausal women [9]. As a result, the coexistence of a carcinoid tumor within an ovarian teratoma is extremely rare in young patients and, to our knowledge, our case represents potentially one amongst the very few that have occurred in women less than 30 years old. 

Optimal therapeutic strategy remains the main challenge regarding ovarian teratomas with TMT. The majority of ovarian mature teratomas with malignant transformations are observed in postmenopausal women, in which a more radical surgical treatment including hysterectomy and bilateral salpingo-oophorectomy may be considered as a reasonable option [2]. However, regarding younger women not having completed their childbearing and especially nulliparous, as in our case, the dilemma between fertility preservation and prevention of further malignancy progression is pended. Despite the fact that local excision of the tumor or unilateral salpingo-oophorectomy seems to be the most reasonable therapeutic option in such cases [2], no consensus has yet been achieved because of the small number of reported cases. However, there are reports of carcinoid tumors arising in mature teratomas treated with a conservative surgical approach, without evidence of recurrence disease after several months of follow-up, implying conservative strategy for young patients who want to preserve their fertility [10]. 

Clinical and histopathologic prognostic factors of the neoplasm represent the basic determinants of the therapeutic strategy in such rare cases without evidence-based guidelines. Cyst wall invasion, intraoperative rupture of the ovarian mass, tumor dissemination, and adhesions are mainly considered as unfavorable prognostic factors [12]. Furthermore, the observation of clinical symptoms and signs such as flushing, edema, diarrhea, abdominal cramps, respiratory distress, and cardiac dysfunction that are caused by the secretion of vasoactive factors from the neuroendocrine cells, which is usually referred to as the “carcinoid syndrome,” may be indicative of a rather aggressive biological behavior [10]. However, in our case, no unfavorable pathologic or clinical signs or symptoms were observed. Therefore, considering the low metastatic potential of the present tumor, the young age, and the nulliparous status of our patient, no further radical surgical approach was decided, while close followup according to the oncologic standards was organized.

In conclusion, despite the weak evidence related to the coexistence of a carcinoid tumor within a mature teratoma in younger patients, it seems that fertility spare surgical approach for women wanting to preserve their genital tract may be a reasonable option. However, further evidence is needed in order to support definitively the option of conservative treatment in such cases without compromising patient's survival.
